# Prognosis following Upper Gastrointestinal Bleeding

**DOI:** 10.1371/journal.pone.0049507

**Published:** 2012-12-12

**Authors:** Stephen E. Roberts, Lori A. Button, John G. Williams

**Affiliations:** College of Medicine, Swansea University, Swansea, United Kingdom; University of Aberdeen, United Kingdom

## Abstract

**Background:**

Upper gastrointestinal (GI) bleeding is one of the most common, high risk emergency disorders in the western world. Almost nothing has been reported on longer term prognosis following upper GI bleeding. The aim of this study was to establish mortality up to three years following hospital admission with upper GI bleeding and its relationship with aetiology, co-morbidities and socio-demographic factors.

**Methods:**

Systematic record linkage of hospital inpatient and mortality data for 14 212 people in Wales, UK, hospitalised with upper GI bleeding between 1999 and 2004 with three year follow-up to 2007. The main outcome measures were mortality rates, standardised mortality ratios (SMRs) and relative survival.

**Results:**

Mortality at three years was 36.7% overall, based on 5215 fatalities. It was highest for upper GI malignancy (95% died within three years) and varices (52%). Compared with the general population, mortality was increased 27-fold during the first month after admission. It fell to 4.3 by month four, but remained significantly elevated during every month throughout the three years following admission.

The most important independent prognostic predictors of mortality at three years were older age (mortality increased 53 fold for people aged 85 years and over compared with those under 40 years); oesophageal and gastric/duodenal malignancy (48 and 32 respectively) and gastric varices aetiologies (2.8) when compared with other bleeds; non-upper GI malignancy, liver disease and renal failure co-morbidities (15, 7.9 and 3.9); social deprivation (29% increase for quintile V vs I); incident bleeds as an inpatient (31% vs admitted with bleeding) and male patients (25% vs female).

**Conclusion:**

Our study shows a high late as well as early mortality for upper GI bleeding, with very poor longer term prognosis following bleeding due to malignancies and varices. Aetiologies with the worst prognosis were often associated with high levels of social deprivation.

## Introduction

Upper gastrointestinal (GI) bleeding is one of the most important high risk emergency disorders in the Western world. In the UK alone, it leads to approximately 25 000 inpatient admissions annually, with a high mortality rate that varies from about 3% to 18% across hospitals each year [1], [2]. The main causes of upper GI bleeds include peptic ulcer, gastritis, oesophagitis, duodenitis, varices, upper GI malignancies and Mallory-Weiss tear [1]–[6]. The contributions of these different aetiologies have changed over time in many countries, for example, following h. pylori. eradication therapy for peptic ulcer and changes over time in alcohol consumption.

Upper GI bleeding has been the subject of many recent audits and other studies that have investigated short term mortality (mainly as an inpatient or at 30 days post admission) [3]–[19], and explored how it is affected by factors such as aetiology, admission to a dedicated bleed unit, management approach, week day of admission and provision of out of hours diagnostic and therapeutic endoscopy services. Almost nothing has been reported about longer term prognosis following upper gastrointestinal bleeding [20].

The main objectives of this study were, firstly, to establish mortality up to three years following hospital admission with upper GI bleeding and, secondly, to determine how it is related to aetiology, co-morbidities and socio-demographic factors, including social deprivation.

## Methods

### Study design

For this study, we used a retrospective cohort design for all people in Wales, UK (population 3.0 million) who were hospitalised with upper GI bleeding between 1999 and 2004, with three year follow up to 2007. The study was based on hospital inpatient data, from the Patient Episode Database for Wales (PEDW), with systematic record linkage to death certificate data from the National Health Service (NHS) Welsh Administrative Register, as part of the Secure Anonymised Information Linkage (SAIL) databank [21]–[23]. As described previously [17], [21], [22], PEDW covers inpatient and day case admissions to all NHS hospitals across 22 local health authorities and has been used as the basis of many previous published studies [17], [23]–[29]. The systematic record linkage of inpatient and mortality data enables deaths following discharge from hospital to be identified and included with in-hospital deaths. In order to obtain information on the causes of the deaths, we also record linked to death certificate data from the Office for National Statistics (causes of death available from January 2003).

### Ethics statement

Ethical approval and patient consent for the study data (Patient Episode Database for Wales and National Health Service Welsh Administrative Register) were not required. This data is publicly available to other researchers. We were advised by the National Research Ethics Service (NRES) that ethical approval and written patient consent were not required as we were using fully anonymised data, which we had obtained approval from the Information Governance Review Panel (IGRP) to use in this study. The IGRP is represented by NRES, the British Medical Association Ethics Advisor, the Caldicott Guardian and NHS Wales Informatics Service.

### Setting

The study covered admissions with upper GI bleeding among people resident in Wales to all NHS hospitals in Wales. Most of the study patients (94.5%) were admitted to the 18 district general hospitals in Wales, the remainder to 93 other NHS hospitals. The private sector in Wales is small and rarely receives emergency admissions. While each of the 18 district general hospitals provide in-hours endoscopy services, three provided out of hours endoscopy services as part of a formal rota during the study period, although this has been shown to have had little impact on 30 day mortality following upper GI bleeding [17]. At the end of the study period, the total population of Wales was 2.98 million. Of these, 2.34 million (78.6%) were aged >18 years and 0.531 million (17.8%) were aged >65 years [30]. Life expectancy was 77.2 years for men and 81.6 years for women [31], and the all cause mortality rate (standardised to the European population) was 718 per 100 000 population [32].

### Inclusion criteria and exclusion criteria

We included adult patients aged ≥18 years who were admitted as an emergency for upper GI bleeding (where upper GI bleeding was recorded as the principal diagnosis on the first episode during the admission). Some upper GI bleeds occur as ‘inpatient bleeds’ following an admission for another disorder, for surgery or for maternities. As described previously [17], we therefore also included emergency, elective and maternity admissions for other causes (when recorded as the principal diagnosis on the first episode) but where upper GI bleeding occurred subsequently (when recorded as the principal diagnosis of a subsequent episode during the admission). The International Classification of Diseases, 10th edition (ICD-10) codes used for upper GI bleeding are listed in Appendix S1. We included each person's first admission with upper GI bleeding after the start of the study period in April 1^st^ 1999, through to March 31^st^ 2004, and followed each of them up for three years, through to the end of the study, March 31^st^ 2007.

### Exposure measures

We investigated the aetiology of the upper GI bleeds using diagnoses recorded in any of the diagnostic fields during the admission (ICD-10 codes are listed in the notes to Table 1). We investigated the following major co-morbidities also using diagnoses recorded in any of the diagnostic fields; circulatory diseases (ICD-10 codes = I00-I99), all malignancies except upper gastrointestinal (C00-C14, C17.2-C97), liver disease (K70-K77), renal failure (N17-N19), chronic obstructive pulmonary disease (COPD; J40-J44) and diabetes (E10-E14). Admissions for these morbidities during three year follow-up were identified using the same ICD-10 codes, where recorded as the principal diagnosis at discharge. Subsequent admissions with upper GI bleeding during follow-up or during the 15 months before the start of the study were identified using the same criteria as for the index study bleed.

**Table 1:** Socio-demographic characteristics of the 14 212 patients hospitalised with upper GI bleeding according to aetiology/main diagnoses.

			Age of patient		Sex of patient		Social deprivation	
Aetiology/main diagnoses *	No. of patients	(% of cases)	Mean age (years)	(95% CI)	% men	(95% CI)	% of patients in the two most deprived quintiles (40% of people)	(95% CI)
Mallory-Weiss tear	894	(6.3%)	47.5	(46.1, 48.9)	64.1%	(60.9%, 67.3%)	51.1%	(47.8%, 54.4%)
Varices – oesophageal	329	(2.3%)	58.9	(57.3, 60.5)	65.7%	(60.3%, 70.8%)	48.9%	(43.4%, 54.4%)
– gastric	30	(0.2%)	62.3	(56.4, 68.2)	66.7%	(47.0%, 83.0%)	53.3%	(34.3%, 72.3%)
– overall	359	(2.5%)	59.2	(57.8, 60.6)	65.8%	(61.1%, 70.2%)	49.3%	(44.5%, 54.1%)
Ulcer – duodenal	1664	(11.7%)	63.5	(62.8, 64.3)	63.8%	(61.4%, 66.1%)	44.5%	(42.1%, 46.9%)
– gastric	1578	(11.1%)	63.4	(62.4, 64.4)	54.5%	(52.0%, 57.0%)	46.8%	(44.3%, 49.3%)
– peptic, unspecified	192	(1.4%)	70.3	(68.0, 72.6)	53.5%	(46.2%, 60.7%)	46.9%	(39.7%, 54.1%)
– gastrojejunal	23	(0.2%)	71.0	(61.3, 80.7)	60.9%	(38.4%, 80.7%)	34.8%	(16.4%, 53.2%)
Malignancy – oesophageal	55	(0.4%)	70.1	(65.6, 74.6)	63.6%	(49.5%, 76.2%)	30.9%	(19.1%, 42.7%)
– gastric/duodenal	92	(0.6%)	75.1	(71.7, 78.5)	69.6%	(59.1%, 78.8%)	50.0%	(39.4%, 60.6%)
– all upper GI	147	(1.0%)	73.2	(71.2, 75.3)	67.4%	(61.1%, 72.9%)	42.9%	(36.9%, 48.8%)
Oesophagitis	1381	(9.7%)	65.8	(64.8, 66.8)	60.5%	(57.9%, 63.1%)	44.9%	(42.3%, 47.5%)
Gastritis & duodenitis	1988	(14.0%)	64.7	(63.9, 65.5)	59.5%	(57.3%, 61.7%)	47.4%	(45.2%, 49.6%)
Other specific diseases of stomach & duodenum, including gastric antral vascular ectasia	148	(1.0%)	71.4	(69.4, 73.4)	48.6%	(40.3%, 57.0%)	42.6%	(34.5%, 50.7%)
Complications of anti-coagulants	101	(0.7%)	74.1	(71.8, 76.2)	48.5%	(38.4%, 58.7%)	46.5%	(36.5%, 56.5%)
Complications of analgesics, antipyretics & anti-inflammatory drugs (AAAs)	405	(2.8%)	70.9	(69.2, 72.6)	48.9%	(43.9%, 53.9%)	38.8%	(34.0%, 43.6%)
Other & unspecified aetiologies	6354	(44.7%)	65.2	(64.7, 65.7)	58.7%	(57.7%, 59.9%)	46.1%	(44.9%, 47.4%)
**All cases**	14 212	(100%)	64.1	(63.8, 64.4)	53.5%	(52.7%, 54.3%)	48.0%	(47.2%, 48.8%)

**Notes**

* The ICD-10 codes used for the aetiology/main diagnoses were as follows:

Mallory-Weiss tear (K22.6), varices – oesophageal (I85), varices – gastric (I86.4), ulcer – duodenal (K26), ulcer – gastric (K25), ulcer – peptic, unspecified (K27), ulcer – gastrojejunal (K28), malignancy – oesophageal (C15), malignancy – gastric/duodenal (C16, C17.0), oesophagitis (K20, K21.1, K22.1), gastritis & duodenitis (K29), other specific diseases of stomach & duodenum, including gastric antral vascular ectasia (K31.8), complications of anti-coagulants (Y44.2), complications of analgesics, antipyretics & anti-inflammatory drugs (Y45).

Social deprivation scores were assigned anonymously using residential postcodes at the Lower Super Output Area (LSOA; average population size of each LSOA = 1560 people) using the Welsh Index of Multiple Deprivation (WIMD) 2005 [33], which is closely compatible with the similar, widely used English Indices of Multiple Deprivation (IMD) [19]. WIMD 2005 comprises seven domains of social deprivation with the following weighting assigned to each domain; income (25% weighting), employment (25%), education (15%), health (15%), access to services (10%), housing (5%) and physical environment (5%). The LSOAs were ranked according to their WIMD 2005 score and were categorised into quintiles, with quintile I representing the 20% of least deprived LSOAs and quintile V the 20% of most deprived LSOAs.

### Outcome measures and statistical analysis

The main outcome measures were mortality rates, standardised mortality ratios (SMRs) and relative survival up to three years following hospitalisation with upper GI bleeding. These included deaths that occurred in hospital during the admission with upper GI bleeding as well as those that occurred following discharge. Mortality rates were calculated using the numbers of deaths (from all causes) as the numerators and the numbers of hospitalised cases as the denominators, expressed as percentages, and were standardised directly using the study population of people admitted with upper GI bleeding. Relative survival and standardised mortality ratios (SMRs) were used to show how survival/mortality in the study patients compared with that in the general population of Wales. Relative survival was calculated as the ratio of the observed survival in the hospitalised patients compared with that in the corresponding (aged and sex matched) general population of Wales.

SMRs were calculated using the indirect method and by using two different control group comparisons. Firstly, by applying the age and sex specific mortality rates in the general adult population of Wales over the eight year study period (total n = 18.2 million) to the numbers of patients hospitalised with upper GI bleeding (n = 14 212) to obtain the expected deaths in the study population, and then by comparing the observed and expected deaths. The second control comparison was made by applying age and sex specific mortality rates, additionally matched for social deprivation and the six major co-morbidities, from the general population of adult patients in primary care data across Wales throughout the eight year study period (total n = 3.75 million), to the study patients. The primary care data was obtained through the SAIL databank [21]–[23], and covers one third of all general practices across Wales during the study period.

To determine factors (socio-demographic, aetiology and co-morbidities) that were most important in affecting prognosis at three years, we used multiple logistic regression. We used a parsimonious stepwise modelling approach to avoid over-fitting and multicollinearity, whereby at each stage terms added and removed from the model were tested for significance, so that the final model comprised only of those terms that had significant independent effect on mortality. Significance was measured at the 5% level, and a bonferroni correction was used to assess multiple comparisons.

## Results

There were 14 212 people admitted with upper GI bleeding from 1999 to 2004. Most (13 309; 94%) presented for admission with upper GI bleeding, 903 (6.4%) occurred as inpatient bleeds, and the majority (7605; 54%) were men. The mean age of the patients was 64.1 years (standard deviation = 20.3 years; range = 18–103 years). Women were older (p<0.001) than men (mean 68.5 years vs 60.4 years).

The most common diagnoses for the bleeds were peptic ulcer (3434; 24%) – including duodenal ulcer (1664; 12%), gastric ulcer (1578; 11%) and unspecified peptic ulcer (192; 1.4%) – followed by gastritis & duodenitis (1988; 14%), oesophagitis (1381; 10%) and Mallory-Weiss tear (894; 6.3%; Table 1).

Table 1 also shows socio-demographic characteristics of the 14 212 patients according to aetiology of the bleed. Mallory-Weiss tear and oesophageal varices aetiologies were most common among younger patients, gastric/duodenal malignancy and complications of anti-coagulants among older patients. Upper GI malignancy and varices were most prevalent among men, while Mallory-Weiss tear and gastric varices tended to be most common among the two most socially deprived quintiles (Table 1). For most aetiologies - and for upper GI bleeds overall - patients were disproportionally among the two most deprived quintiles (>40% of patients).

### Mortality up to three years following hospital admission

At three years after admission, there were 5215 deaths from all causes (mortality rate = 36.7%; Table 2). Mortality as an inpatient was 9.7% and mortality at 30 days was 10.0%. Age specific mortality increased from 1.1% among people aged 18–24 years to 72% for people 85+ years (Table 2). Mortality ranged from 13% for Mallory-Weiss tear to 34% for gastritis & duodenitis, 38–41% for duodenal ulcer, gastric ulcer and oesophagitis, 52% for varices and 95% for upper GI malignancy (Table 2). Mortality was also higher for bleeds that occurred as inpatients (54%) than for bleeds presenting at admission (36%).

**Table 2:** Numbers of patients, numbers of deaths at three years, crude mortality rates and standardised mortality ratios (SMRs) for people hospitalised with upper GI bleeding according to prognostic factors.

		Three year follow-up					
				Comparison with age and sex matched general population		Comparison with age, sex, deprivation and co-morbidity matched GP population	
	No. of patients	No. of deaths	Crude mortality rate (%)	SMR	95% CI)	SMR	(95% CI
**All cases**	14 212	5215	36.7%	2.6	(2.5, 2.7)	2.1	(2.0, 2.2)
**Age group (years)**							
18–24	656	7	1.1%	5.7	(2.3, 10.8)	10.5	(4.2, 19.7)
25–34	993	42	4.2%	14.2	(10.2, 18.9)	7.0	(5.0, 9.3)
35–44	1288	133	10.3%	22.8	(19.1, 26.8)	9.3	(7.8, 11.0)
45–54	1418	248	17.5%	16.8	(14.8, 18.9)	7.8	(6.9, 8.9)
55–64	1628	439	27.0%	9.9	(9.0, 10.8)	6.5	(5.9, 7.1)
65–74	2595	972	37.5%	5.0	(4.7, 5.3)	4.1	(3.8, 4.4)
75–84	3536	1862	52.7%	2.7	(2.6, 2.8)	2.2	(2.1, 2.3)
85+	2098	1512	72.1%	1.5	(1.4, 1.5)	1.2	(1.2, 1.3)
**Sex**							
Men	7605	2633	34.6%	2.9	(2.8, 3.0)	2.1	(2.0, 2.2)
Women	6603	2582	39.1%	2.4	(2.3, 2.5)	2.2	(2.1, 2.3)
**Aetiology/main diagnoses**							
Mallory-Weiss tear	894	119	13.3%	2.3	(1.9, 2.7)	1.8	(1.5, 2.1)
Varices – oesophageal	329	167	50.8%	7.6	(6.5, 8.8)	5.7	(4.9, 6.6)
– gastric	30	21	70.0%	7.2	(4.5, 10.5)	5.4	(3.4, 7.2)
– overall	359	188	52.4%	7.5	(6.5, 8.6)	5.7	(4.9, 6.5)
Ulcer – duodenal	1664	638	38.3%	2.5	(2.3, 2.7)	2.0	(1.9, 2.2)
– gastric	1578	648	41.1%	2.6	(2.4, 2.8)	2.1	(1.9, 2.3)
– peptic, unspecified	192	77	40.1%	2.4	(1.9, 3.0)	2.0	(1.5, 2.4)
– gastrojejunal	23	12	52.2%	3.1	(1.6, 5.0)	2.4	(1.2, 4.0)
Malignancy – oesophageal	55	52	94.5%	7.2	(5.3, 9.2)	5.8	(4.3, 7.5)
– gastric/duodenal	92	87	94.6%	5.2	(4.1, 6.3)	4.1	(3.3, 5.1)
– all upper GI	147	139	94.6%	5.8	(4.9, 6.8)	4.6	(3.9, 5.4)
Oesophagitis	1381	527	38.2%	2.6	(2.4, 2.9)	2.1	(2.0, 2.3)
Gastritis & duodenitis	1988	676	34.0%	2.6	(2.4, 2.8)	2.1	(2.0, 2.3)
Other specific diseases of stomach & Duodenum including gastric antral vascular ectasia	148	62	41.9%	2.3	(1.8, 3.0)	1.9	(1.5, 2.4)
Complications of anti-coagulants	101	55	54.5%	3.0	(2.2, 3.8)	2.5	(1.9, 3.2)
Complications of analgesics, antipyretics & anti-inflammatory drugs (AAAs)	405	139	34.3%	1.9	(1.6, 2.2)	1.6	(1.3, 1.8)
Other & unspecified aetiologies	6354	2370	37.3%	2.7	(2.6, 2.8)	2.2	(2.1, 2.3)
**Major co-morbidities** *							
Circulatory diseases	4660	2443	52.4%	2.8	(2.7, 2.9)	2.3	(2.2, 2.4)
Malignancies (all except upper GI)	748	667	89.2%	5.2	(4.8, 5.6)	4.1	(3.8, 4.5)
Liver disease	590	355	60.2%	13.5	(12.2, 15.0)	10.0	(9.0, 11.1)
COPD	504	322	63.9%	3.5	(3.1, 3.8)	2.8	(2.5, 3.1)
Diabetes	1226	625	51.0%	3.2	(3.0, 3.5)	2.6	(2.4, 2.8)
Renal failure	403	307	76.2%	3.8	(3.4, 4.2)	3.0	(2.7, 3.4)
**Social deprivation**							
I (most affluent quintile)	2045	795	38.9%	2.3	(2.2, 2.5)	1.9	(1.8, 2.0)
II	2404	916	38.1%	2.5	(2.3, 2.6)	2.0	(1.9, 2.2)
III	2941	1112	37.8%	2.5	(2.4, 2.6)	2.1	(1.9, 2.2)
IV	3130	1131	36.1%	2.7	(2.5, 2.8)	2.2	(2.1, 2.3)
V (most deprived quintile)	3692	1261	34.2%	3.1	(2.9, 3.2)	2.5	(2.4, 2.6)
**Inpatient bleed**							
No	13 309	4728	35.5%	2.6	(2.5, 2.6)	2.1	(2.1, 2.2)
Yes	903	487	53.9%	3.1	(2.8, 3.3)	2.5	(2.3, 2.7)
**Previous admissions with upper GI bleeding in the 15 months before the study**							
None	13 851	5062	36.5%	2.6	(2.5, 2.7)	2.1	(2.1, 2.2)
1	289	132	45.7%	3.5	(2.9, 4.1)	2.8	(2.3, 3.3)
2	39	16	41.0%	3.9	(2.2, 6.0)	3.1	(1.8, 4.8)
3+	33	5	15.2%	3.7	(1.2, 7.7)	2.9	(0.9, 6.0)
**Admissions with upper GI bleeding**							
**during three year follow-up**							
1	13 019	4736	36.4%	2.6	(2.5, 2.6)	2.1	(2.0, 2.2)
2	985	392	39.8%	3.1	(2.8, 3.4)	2.6	(2.3, 2.8)
3	154	65	42.2%	3.8	(2.9, 4.7)	3.2	(2.4, 4.0)
4+	54	22	40.7%	4.5	(2.8, 6.6)	3.7	(2.3, 5.4)
**Admissions for major morbidities during three year follow-up**							
Circulatory diseases - No	12 935	4539	35.1%	2.5	(2.4, 2.6)	2.1	(2.1, 2.2)
- Yes	1277	676	52.9%	4.1	(3.8, 4.5)	3.3	(3.0, 3.5)
All malignancies except upper GI - No	13 601	4721	34.7%	2.5	(2.4, 2.6)	2.0	(2.0, 2.1)
- Yes	611	494	80.9%	5.3	(4.9, 5.8)	4.2	(3.9, 4.6)
Liver disease - No	13 931	5060	36.3%	2.6	(2.5, 2.6)	2.1	(2.0, 2.1)
- Yes	281	155	55.2%	18.5	(15.7, 21.5)	13.1	(11.1, 15.2)
COPD - No	14 046	5117	36.4%	2.6	(2.5, 2.7)	2.1	(2.1, 2.2)
- Yes	166	98	59.0%	3.7	(3.0, 4.4)	2.9	(2.4, 3.5)
Diabetes - No	14 107	5171	36.7%	2.6	(2.5, 2.7)	2.1	(2.1, 2.2)
- Yes	105	44	41.9%	4.0	(2.9, 5.2)	3.2	(2.3, 4.2)
Renal failure - No	14 126	5155	36.5%	2.6	(2.5, 2.7)	2.1	(2.1, 2.2)
- Yes	86	60	69.8%	6.6	(5.0, 8.4)	4.9	(3.7, 6.2)

**Notes**

* The ICD-10 codes used for the co-morbidities and the admissions for morbidities were as follows; circulatory diseases (ICD-10 = I00-I99), all malignancies except upper GI (C00-C14, C17.2-C97), liver disease (K70-K77), COPD (J40-J44), diabetes (E10-E14) and renal failure (N17-N19).

Age adjusted mortality was 27% higher among women than men (95% CI = 22–33%). Age and sex adjusted mortality was 28% higher (95% CI = 13–45%) for the most deprived quintile of patients than the most affluent quintile. Mortality was also increased for patients who had previous admissions for upper GI bleeding in the 15 months before the study period, or who had subsequent admissions with upper GI bleeding or for other major disorders during the three year follow-up (Table 2).

**Figure 1:**
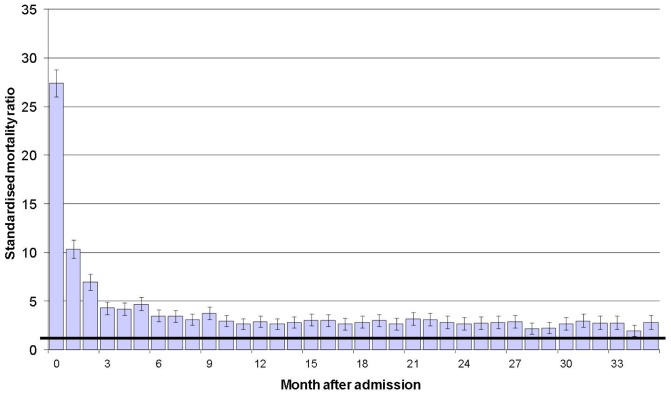
Standardised mortality ratios (SMRs) at monthly intervals up to three years following hospital admission with upper GI bleeding. Vertical bars represent 95% confidence intervals. The horizintal line in bold respresents mortality in the general population (SMR = 1).

Compared with the age and sex matched general population (SMR = 1.0), all cause mortality was increased 27-fold during the first month after admission (Figure 1). This increased risk fell to 4.3 during month four, but mortality remained significantly elevated during every month throughout the three years following admission.

Mortality at three years was increased when compared with the age and sex matched general Welsh population (SMR = 2.61), and also when compared with the age, sex, social deprivation and co-morbidity matched Welsh population from primary care (SMR = 2.14; Table 2). Mortality at three years was also increased for all aetiologies, most strongly for oesophageal varices (SMR = 7.6 compared with the general population), followed by oesophageal malignancies and gastric varices (both SMRs = 7.2) and most weakly for Mallory-Weiss tear (SMR = 2.3; Table 2).

**Figure 2:**
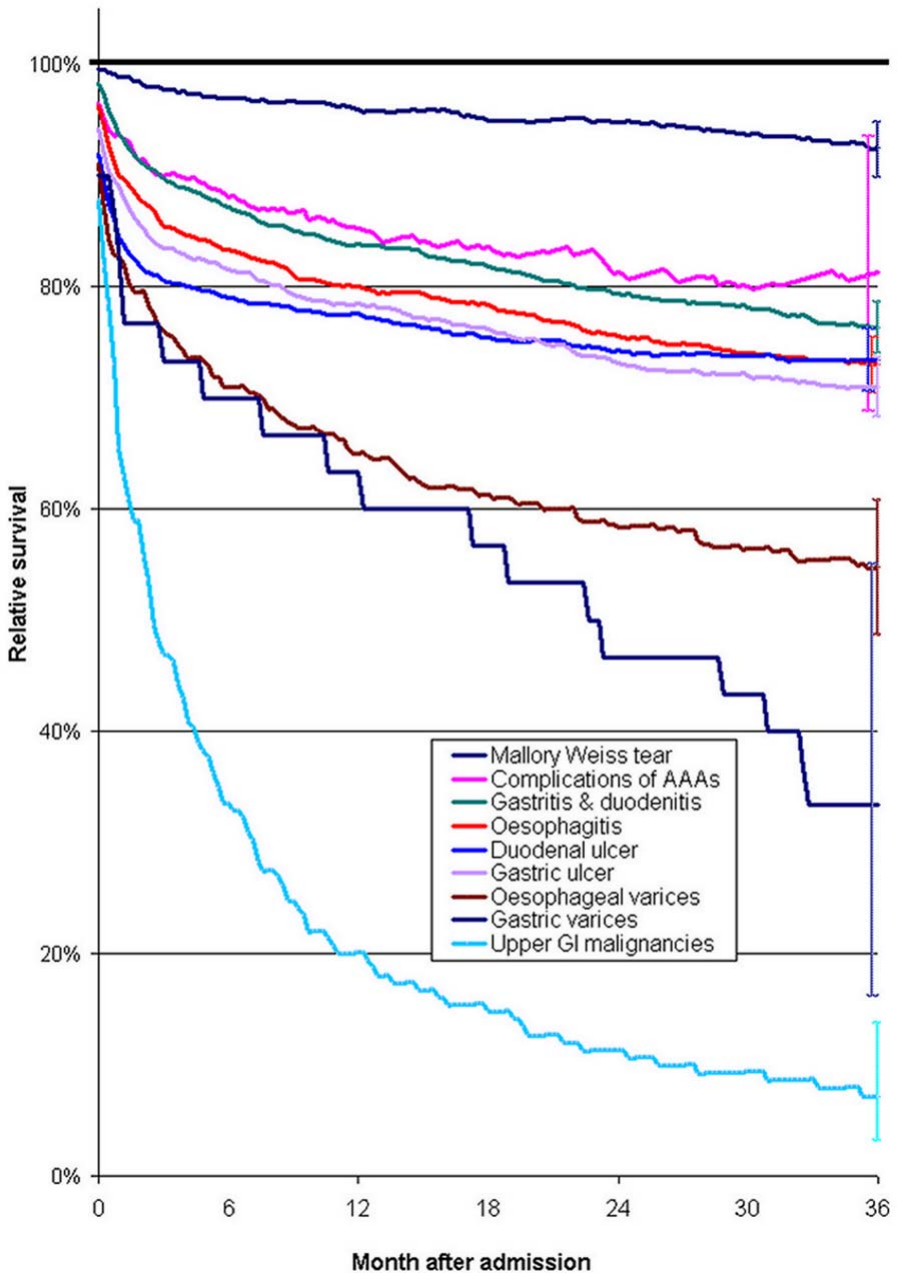
Relative survival during the three years following hospital admission with upper GI bleeding, compared with that in the general population, according to the aetiology of the bleed. The horizontal line in bold at 100% denotes relative survival in the general population. Vertical bars on the far right represent 95% confidence intervals. Relative survival is standardised for age group and sex. Complications of AAAs = Complications of analgesics, antipyretics & anti-inflammatory drugs.

Relative survival - compared with the general population - was best for Mallory-Weiss tear aetiology and worst for malignancies and varices (Figure 2). Relative survival was substantially higher for duodenal ulcer than for gastric ulcer during the first few months after admission, but was worse for gastric ulcer in the longer term. For ‘complications of analgesics, antipyretics & anti-inflammatory drugs’ and duodenal ulcer aetiologies, survival almost levelled off to that in the general population, but for the other aetiologies, it remained worse throughout the three year follow up (Figure 2).

Based on underlying cause of death, the causes of 1920 of these deaths from January 2003 onwards were upper GI bleeding (128 deaths; 6.7%), ischaemic heart disease (283; 14.7%), stroke (185; 9.6%), other circulatory diseases (156; 8.1%), GI malignancies (182; 9.5%), liver disease (114; 5.9%), other GI diseases (77; 4.0%), other malignancies (225; 11.7%), pneumonia (133; 6.9%), COPD (66; 3.4%) and various other disorders (371; 19.3%).

### Predictors of mortality at three years

Using multivariate analysis (Table 3), the most important predictors of mortality at three years, with significant independent effect were as follows. Firstly, the age of the patient. There was a 53-fold increased risk of mortality for people aged 85+ years when compared with patients aged <40 years (Table 3). Secondly, aetiology of the bleed. There were significantly increased risks of mortality of 48 for oesophageal malignancy, 32 for gastric/duodenal malignancy and 2.8 for gastric varices aetiologies, when compared with all other & unspecified bleeds. There were also significantly reduced risks of 46% for Mallory-Weiss tear, 31% for ‘complications of analgesics, antipyretics & anti-inflammatory drugs’ and 18% for gastritis & duodenitis aetiologies. Third, patient co-morbidities. There were significantly increased risks of 15 for patients with non-upper GI malignancies, 7.9 for liver disease and 3.9 for renal failure co-morbidities when compared with patients without these co-morbidities. Fourth, ‘inpatient bleeds’ that occur as an inpatient, with a 31% increased risk compared with bleeds at admission. Fifth, social deprivation, with a 29% increased risk for patients in the most deprived quintile V compared with the most affluent quintile I. Finally, the sex of the patient, with a 25% increased risk for men compared with women. When using a bonferroni correction for the multiple comparisons (p = 0.0031), all of these factors remained significant except gastric varices.

**Table 3:** Multivariate analysis showing prognostic factors with significant and independent influence on mortality at three years following hospital admission with upper GI bleeding.

Prognostic factor ‡	Adjusted Odds ratio *	(95% CI) †		P-value ‡
**Age group**				<0.001
<40	1.00	Ref		
40–44	1.77	(1.26, 2.48)		
45–49	2.39	(1.76, 3.24)		
50–54	3.26	(2.47, 4.31)		
55–59	3.94	(2.99, 5.18)		
60–64	5.64	(4.36, 7.30)		
65–69	7.01	(5.50, 8.94)		
70–74	10.1	(8.00, 12.7)		
75–79	14.5	(11.6, 18.2)		
80–84	23.1	(18.4, 29.0)		
85+	52.9	(42.2, 66.4)		
**Sex**				<0.001
Women	1.00	Ref		
Men	1.25	(1.15, 1.37)		
**Social deprivation**				0.001
I (most affluent quintile)	1.00	Ref		
II	1.06	(0.92, 1.23)		
III	1.05	(0.92, 1.21)		
IV	1.14	(0.99, 1.31)		
V (most deprived quintile)	1.29	(1.12, 1.47)		
**Inpatient bleed**				
No	1.00	Ref		
Yes	1.31	(1.12, 1.54)		0.001
**Aetiology/main diagnoses**				
All other & unspecified aetiologies	1.00	Ref		
Mallory-Weiss tear	0.54	(0.43, 0.69)		<0.001
Varices – gastric	2.80	(1.12, 7.01)		0.028
Malignancy – oesophageal	48.0	(14.3, 160)		<0.001
Malignancy – gastric/duodenal	32.2	(12.8, 80.8)		<0.001
Gastritis & duodenitis	0.82	(0.73, 0.92)		0.001
Complications of analgesics, antipyretics & anti-inflammatory drugs (AAAs)	0.69	(0.54, 0.87)		0.002
**Major co-morbidities**				
None	1.00	Ref		
Circulatory diseases	1.49	(1.36, 1.62)		<0.001
Malignancies (all except upper GI)	15.4	(11.8, 20.1)		<0.001
Liver disease	7.89	(6.44, 9.68)		<0.001
COPD	2.20	(1.79, 2.71)		<0.001
Diabetes	1.35	(1.18, 1.55)		<0.001
Renal failure	3.93	(3.02, 5.12)		<0.001

**Notes**

* Odds ratios for sex are adjusted for age group, odds ratios for social deprivation, inpatient bleed, aetiology/main diagnoses, and major c-morbidities are adjusted for age group and sex

† Ref = Reference category

‡ When applying the Bonferroni correction to adjust for the multiple comparisons (p = 0.0031), all terms in this model remained statistically significant with the exception of gastric varices (which was based on only 30 cases). None of the two-way interaction effects between these terms were significant when applying the Bonferroni correction (p = 0.0004) or at the p = 0.001 level.

## Discussion

The aim of this study was to establish, for the first time, longer term prognosis for people hospitalised with upper GI bleeding and its relationship with aetiology, co-morbidities and socio-demographic factors. We found that overall mortality at three years following admission with upper GI bleeding was high at 36.7%. It was highest for upper GI malignancies (95%) and varices (52%; particularly gastric varices, 70%) aetiologies. Compared with the general population, mortality was increased 27-fold during the first month after admission, this SMR fell to 4.3 after three months, but it remained elevated during every month in the three years following admission. Mortality was associated with social deprivation, with highest rates among people in the most deprived quintile. Admission with upper GI bleeding thus carries both a high early and late mortality.

Major strengths of this study are that it provides new evidence on longer term prognosis for upper GI bleeding. Secondly, it is one of the largest studies of upper GI bleeding, covering more than 14 000 people who were hospitalised. Thirdly, it is based on systematic record linkage to enable reliable longer term follow-up and to identify deaths that occur following discharge from hospital. The record linkage, principal inpatient diagnoses and ascertainment of mortality have been validated previously and have been shown to be respectively >99.8%, >90% and >98% accurate [21], [29].

Limitations of the study are, firstly, the administrative inpatient data lacks detailed information about disease history, pathology, severity of bleeding and treatment, although some of these factors are of less importance for this study of mortality in the longer term following admission. The measures of prognosis reported here are those from the index admission with upper GI bleeding and we recognise that some of these may occur late in the natural history of upper GI disease. However, since 83% of the study patients were hospitalised once only with upper GI bleeding during the five year inpatient period studied, this should affect a minority of cases.

As in other recent studies that have used national administrative inpatient data [14], [17]–[19], the recording of aetiologies and major co-morbidities would be incomplete in some cases. As per convention in most studies, deaths from all causes were included when investigating mortality rates, as deaths specifically from upper GI bleeding accounted for only a small minority of all deaths, which has been found for many other conditions [34].The study was restricted to NHS hospitals, although the private sector is small and receives few emergencies for upper GI bleeding. Although it is also possible that we may have missed some upper GI bleeds through the limitations of hospital and ICD coding, and included some lower GI bleeds, these discrepancies should be small [17]. The longer term mortality follow-up to three years – instead of the usual inpatient or 30 days – was used to investigate the prognostic impact of aetiology, co-morbidity and socio-demographic factors – rather than treatment and hospital-related factors, which are usually of less relevance beyond the acute phase and inpatient stay. Our cohort should not be affected substantially by attrition over the three year follow-up, as migration out of Wales was about 0.3% per annum during 2005 and 2006, compared with 0.5% immigration [35], and is lower among older age groups who are most frequently affected by upper GI bleeds.

Early mortality for upper GI bleeding (as an inpatient or at 30 days) has been shown to vary according to aetiology from <5% for Mallory-Weiss tear to about 10% for peptic ulcer, 15–25% for varices and >20% for upper GI malignancies [3]–[6], [17]. There was extremely poor prognosis (95% mortality at three years) for upper GI malignancies. We also found that late mortality is very high for varices (52%) - which is consistent with other studies that have reported dismal prognosis for decompensated liver cirrhosis [36]–[40]. Although relatively uncommon (30 cases), the higher mortality for gastric varices rather than for oesophageal varices may indicate more severe portal hypertension and bleeding, as well as greater difficulty in securing haemostasis through therapeutic endoscopy.

Our inpatient mortality rate of 9.7% compares with rates of 4.6%, 9.4%, 10.3% and 15.2% from four previous single centre studies in Wales in recent years [2], and with 10.0% in the recent UK audit [3]. The aetiology distribution of the diagnosed bleeds is also broadly comparable with these previous studies. Previous studies, including ours, have reported no significant differences in early mortality according to social deprivation [6], [17], [19]. At three year follow up, we found significantly higher mortality among the most deprived quintile of patients than the most affluent. This apparent widening of mortality differentials across social groups with longer durations of follow-up after admission reflects general social inequalities in health outcomes. The higher age adjusted mortality among men - compared with women - reflects a higher proportion of highest risk bleeds from varices and malignancies among men than women. The fact that mortality was elevated when a co-morbidity-matched comparison was made with patients from primary care, suggests that the co-morbidities among hospitalised upper GI bleed patients are much more severe than among primary care patients, and would also reflect that a very small minority of high risk people are not registered with GPs.

Upper GI bleeding is one of the most important emergency disorders with high rates of mortality during the acute phase. When compared with the general population, we found that mortality was increased 27-fold during the first month after admission, which is comparable with that found in a study of acute pancreatitis (30-fold increase) [41]. After three months, the increased risk of mortality had fallen to 4.3, but it remained elevated during each month of the three years after admission. This longer term increased risk of mortality is partly due to very poor prognosis for malignancy and variceal aetiologies, although it also reflects an impact of high levels of social deprivation and chronic co-morbid disease among people with upper GI bleeding. Our findings are broadly concordant with the existing but very limited evidence for late mortality. For example, our mortality rate of 41% at three years for peptic ulcer bleeds compares with 29% reported for people aged 60 years and over in the Nottingham region at (an average of) 34 months follow-up [20].

Survival over the three years was substantially poorer than in the general population for most aetiologies of bleeding, with the possible exception of ‘complications of analgesics, antipyretics & anti-inflammatory drugs’ and duodenal ulcers, which were both less prevalent among deprived quintiles than most of the other aetiologies. Relative survival was worse for duodenal ulcer than for gastric ulcer in the first few months after admission, but it was better than for gastric ulcer bleeds in the longer term. This finding is consistent with a large single-centre study of surgery for peptic ulcer which found increased longer term mortality for gastric ulcer but not for duodenal ulcer [42].

The most important predictors of poor prognosis at three years were older age groups, malignancy and gastric variceal aetiologies, inpatient bleeds, male patients, high levels of social deprivation and the following co-morbidities; non-upper GI malignancies, liver disease and renal failure. These show strong similarities with the Rockall score (which includes older age groups, shock, renal failure, liver failure, metastatic cancer, malignancy aetiology and evidence of bleeding at endoscopy as the strongest predictors of poor prognosis in the short term) [43], although we were unable to investigate shock and evidence of bleeding at endoscopy accurately. Our findings also suggest that social deprivation is an important independent predictor of prognosis in the longer term following admission.

## Supporting Information

Appendix S1 **ICD-10 codes used for upper GI bleeding.**
(DOC)
