# Supplementary material for: Prognosis following Upper Gastrointestinal Bleeding
Source: PLoS One. 2012 Dec 12;7(12):e49507. doi: 10.1371/journal.pone.0049507 (PMC3520969; doi:10.1371/journal.pone.0049507)
Supplement: Appendix S1 — ICD-10 codes used for upper GI bleeding. (DOC) [file pone.0049507.s001.doc]

**Appendix ICD-10 codes used for upper GI bleeding:**

K22.6 Gastro-oesophageal laceration-haemorrhage syndrome (Mallory-Weiss Syndrome)

K22.8 Other specified diseases of oesophagus: Haemorrhage of oesophagus NOS

K25.0 Gastric ulcer - Acute with haemorrhage

K25.2 Gastric ulcer - Acute with both haemorrhage and perforation

K25.4 Gastric ulcer - Chronic or unspecified with haemorrhage

K25.6 Gastric ulcer - Chronic or unspecified with both haemorrhage and perforation

K26.0 Duodenal ulcer - Acute with haemorrhage

K26.2 Duodenal ulcer - Acute with both haemorrhage and perforation

K26.4 Duodenal ulcer - Chronic or unspecified with haemorrhage

K26.6 Duodenal ulcer - Chronic or unspecified with both haemorrhage and perforation

K27.0 Peptic ulcer, unspecified - Acute with haemorrhage

K27.2 Peptic ulcer, unspecified - Acute with both haemorrhage and perforation

K27.4 Peptic ulcer, unspecified - Chronic or unspecified with haemorrhage

K27.6 Peptic ulcer, unspecified - Chronic or unspecified with both haemorrhage and perforation

K28.0 Gastrojejunal ulcer – Acute with haemorrhage

K28.2 Gastrojejunal ulcer – Acute with both haemorrhage and perforation

K28.4 Gastrojejunal ulcer – Chronic or unspecified with haemorrhage

K28.6 Gastrojejunal ulcer – Chronic or unspecified with both haemorrhage and perforation

K29.0 Acute haemorrhagic gastritis

I85.0 Oesophageal varices with bleeding

K92.0 Haematemesis

K92.1 Melaena

K92.2 Gastrointestinal haemorrhage, unspecified, unless present with a secondary or subsidiary

diagnosis of lower gastrointestinal disease (C17.1-C21, K50-K52, K55-K57 or K60-K63)
